# Mass Spectrometry-Based Quantitative Metabolomics Revealed a Distinct Lipid Profile in Breast Cancer Patients

**DOI:** 10.3390/ijms14048047

**Published:** 2013-04-12

**Authors:** Yunping Qiu, Bingsen Zhou, Mingming Su, Sarah Baxter, Xiaojiao Zheng, Xueqing Zhao, Yun Yen, Wei Jia

**Affiliations:** 1 Center for Translational Medicine and Department of Endocrinology and Metabolism, Shanghai Jiao Tong University Affiliated Sixth People’s Hospital, Shanghai 200233, China; E-Mails: qyp29@163.com (Y.Q.); joyzheng999@gmail.com (X.Z.); 2 University of Hawaii Cancer Center, Honolulu, HI 96813, USA; 3 Department of Medical Oncology and Therapeutic Research, City of Hope National Medical Center, Duarte, CA 91010, USA; E-Mails: bingsenzhou@gmail.com (B.Z.); yyen@coh.org (Y.Y.); 4 David H. Murdock Research Institute, North Carolina Research Campus, Kannapolis, NC 28081, USA; E-Mails: johnsonsu2008@gmail.com (M.S.); sbaxter@dhmri.org (S.B.); 5 Department of Nutrition, University of North Carolina at Greensboro, North Carolina Research Campus, Kannapolis, NC 28081, USA; E-Mail: x_zhao4@uncg.edu

**Keywords:** breast cancer, lipids, metabolomics/metabonomics, plasma

## Abstract

Breast cancer accounts for the largest number of newly diagnosed cases in female cancer patients. Although mammography is a powerful screening tool, about 20% of breast cancer cases cannot be detected by this method. New diagnostic biomarkers for breast cancer are necessary. Here, we used a mass spectrometry-based quantitative metabolomics method to analyze plasma samples from 55 breast cancer patients and 25 healthy controls. A number of 30 patients and 20 age-matched healthy controls were used as a training dataset to establish a diagnostic model and to identify potential biomarkers. The remaining samples were used as a validation dataset to evaluate the predictive accuracy for the established model. Distinct separation was obtained from an orthogonal partial least squares-discriminant analysis (OPLS-DA) model with good prediction accuracy. Based on this analysis, 39 differentiating metabolites were identified, including significantly lower levels of lysophosphatidylcholines and higher levels of sphingomyelins in the plasma samples obtained from breast cancer patients compared with healthy controls. Using logical regression, a diagnostic equation based on three metabolites (lysoPC a C16:0, PC ae C42:5 and PC aa C34:2) successfully differentiated breast cancer patients from healthy controls, with a sensitivity of 98.1% and a specificity of 96.0%.

## 1. Introduction

According to the American Cancer Society estimation, breast cancer remains to be one of the most commonly diagnosed and death-related cancers in women in the United States [1]. In 2012, about 226,870 females in the U.S. were diagnosed with breast cancer, which represents 29% of all newly diagnosed female cancer patients. Breast cancer was estimated to cause more than 39,000 deaths in the U.S. in the past year, ranking it as the second leading cause of cancer death in women [1]. Early diagnosis can significantly increase long-term survival rates for breast cancer [2]. Currently, mammography is the most acceptable and effective screening procedure for the detection of breast cancer and was recommended by the U.S. Preventive Services Task Force (USPSTF) to women over 40 years old [3]. However, because of the high false positive rate of this screen, the USPSTF revised their recommendation to a reduced frequency of mammogram screening in 2009 [4]. Other imaging techniques, such as ultrasonography and magnetic resonance imaging, have also been used in breast cancer screening. Unfortunately, even with the inclusion of these imaging techniques, about 20% of breast cancer patients still cannot be detected [5]. Plasma (or serum) biomarkers (such as antigens and protein patterns) are promising [6,7]; however, they are still far from clinical use. Some tumor markers, such as CA15.3 and CA27.29, are recommended only for therapeutic monitoring, but not screening [8]. Therefore, new effective biomarkers for breast cancer screening that can be used individually or in combination with other existing methods are urgently needed.

In addition to genetic and proteomic variations, cancer cells have been shown to express distinct metabolic variations compared with normal cells [9]. The advanced metabolomics technology based on mass spectrometry (MS) and nuclear magnetic resonance (NMR) have shown great potential in finding biomarkers with cancer cells or clinical samples [10–14]. To support fast proliferation of cancer cells, increased lipogenesis is believed to be a characteristic metabolic feature of many types of cancer cells [15]. The association between plasma (or serum) lipids (such as total cholesterol, high density lipoprotein and triglycerides) and the risk of breast cancer has also been reported in various studies [16,17]. The advent of advanced profiling technology using MS enables a targeted analysis and simultaneous quantitation of hundreds of lipids in a given biological specimen. Results from MS-based analyses of distinct lipid compositions in breast cancer tissues compared with levels in normal ones were recently reported, revealing a high correlation between lipid metabolism (such as PC (phosphatidylcholine) (14:0/16:0) level) and the tumor grade or estrogen receptor (ER) status [15]. We hypothesized that the variations in lipid expression in tumor tissues would result in a unique lipid profile in the plasma of breast cancer patients, which can be detected with the targeted and quantitative metabolomics approach. Detected differentiating plasma lipids may be useful as new biomarkers for early screening of breast cancer.

In this study, we used a quantitative and targeted metabolomics approach with electrospray ionization tandem mass spectrometry (ESI-MS/MS) to analyze plasma samples from 55 breast cancer patients and 25 healthy controls. The aim of this study was to identify potential diagnostic biomarkers and improve the understanding of lipid metabolism in breast cancer.

## 2. Results and Discussion

### 2.1. Multivariate Analysis

**Figure 1:**
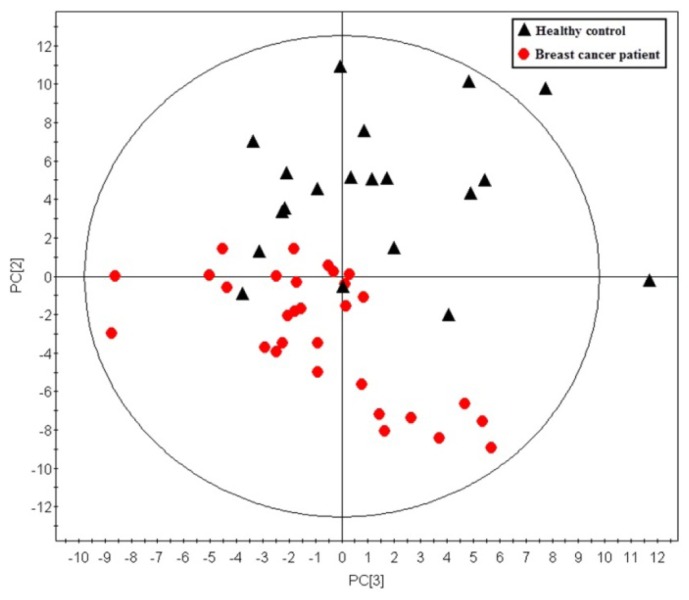
Principle component analysis (PCA) scores plot discriminating the metabolic profiles in plasma of breast cancer patients and those in healthy controls in the training dataset. Each red dot represents one breast cancer patient, while each black triangle represents one healthy control (five components model: R2X = 0.643, Q2 = 0.416, R2X1 = 0.261, R2X2 = 0.165, R2X3 = 0.101).

**Figure 2:**
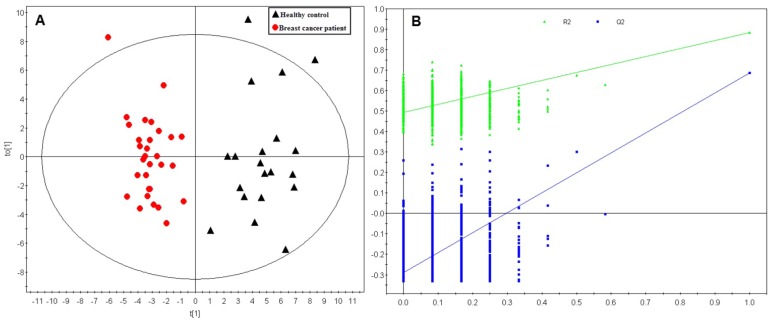
Orthogonal partial least squares-discriminant analysis (OPLS-DA) scores plot and permutation test for the model discriminating plasma samples from breast cancer patients and healthy controls in the training dataset. (**A**) OPLS-DA scores plot. The model parameters were: R2Xcum = 0.464, R2Ycum = 0.884, Q2 = 0.756. Each red dot represents one breast cancer patient, while each black triangle represents one healthy control; (**B**) A 999-times permutation test for the corresponding model. The *Y*-axis intercepts were: R2 (0, 0.498), Q2 (0, −0.295).

A total of 146 metabolites were analyzed in this study. Using those metabolites, an unsupervised principle component analysis (PCA) was first performed on the training dataset. A five component PCA model was obtained with the parameters as: R2X = 0.643, Q2 = 0.416. A clear separation between sample groups was observed, with most of the healthy controls scattering at the top of the plot, while most of the breast cancer samples were scattered across the bottom half (Figure 1). The unsupervised PCA model revealed the general metabolic information between breast cancer patients and healthy controls. To further specify the metabolic variations associated with cancer morbidity, a supervised orthogonal partial least squares-discriminant analysis (OPLS-DA) model was established with one predictive component and two orthogonal components (R2X = 0.464, R2Y = 0.884, Q2 = 0.756). As shown in Figure 2A, a clear separation was obtained in the scores plot, with all the breast cancer patients in the left half and healthy controls in the right half. To further validate the established model, a 999-time permutation test was performed for the corresponding model. The result showed that all the R2 and Q2 values in the permutation test were lower than the original ones. The *Y*-axis intercept for Q2 is below zero (Q2 intercept (0, −0.295)). These results validate the current supervised model.

### 2.2. Differentiating Metabolites Identification

Based on the variable importance in the projection (VIP) values in the OPLS-DA model, 46 metabolites were selected with VIP > 1. Univariate analysis using a Student’s *t*-test was also performed for selection of differentiating metabolites. After a false discovery rate (FDR) test with a classical one-stage method, 39 metabolites were selected with adjusted *p*-values less than 0.05 (Table 1). Those 39 differentiating metabolites included 10 lysophosphatidylcholines (lysoPCs), 23 glycerophosphocholines (PCs, nine PC aas and 14 PC aes), five sphingomyelins (SMs) and 1 acylcarnitine. We further compared the concentrations of these 39 differentiating metabolites in breast cancer patients with Body Mass Index (BMI) < 25 (*n* = 16) and BMI > 30 (obese patients, *n* = 21). Only five among these 39 metabolites were significantly different between patients with normal weight and obese subjects in the student’s *t*-test. These five metabolites were excluded from our diagnostic equation. After FDR adjustment, no metabolites showed a significant difference with a *q*-value lower than 0.05.

### 2.3. Diagnostic Equation

**Figure 3:**
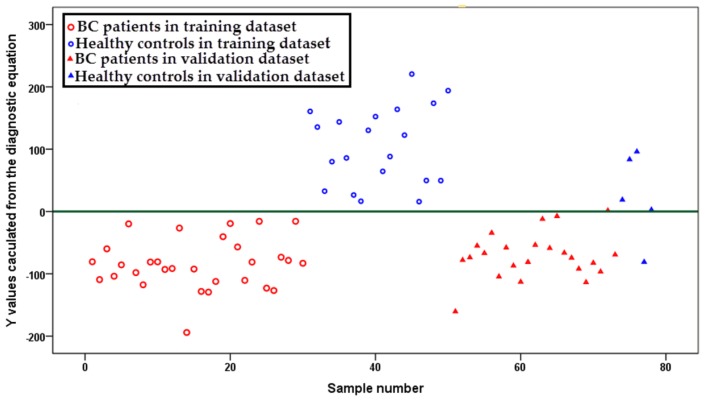
Scatter plot for *y*-values calculated from an established breast cancer *versus* healthy control diagnostic equation. Samples in blue represent healthy controls, while samples in red represent breast cancer patients. Samples represented by circles indicate the training samples and triangles for the validation ones.

To establish a simple diagnostic equation for identifying breast cancer patients from healthy controls, the 39 differentiating metabolites selected from training dataset were imported into SPSS for logical regression. The result indicated that the concentrations of three metabolites (lysoPC a C16:0, PC ae C42:5 and PC aa C34:2) significantly affected the diagnostic result between breast cancer patients and healthy controls. Using the concentrations of these three metabolites, a diagnostic equation of *y* = lysoPC a C16:0 × 1.034 + PC ae C42:5 × 44.248 − PC aa C34:2 × 0.585 − 37.002 was established. *Y*-values for each sample (both in the training and validation dataset) were calculated. A scatter plot demonstrated that almost all *y*-values obtained from breast cancer samples were lower than zero, while the *y*-values from healthy controls were higher than zero (except one breast cancer sample and one healthy control in the validation dataset, Figure 3), with a sensitivity of 98.1%, a specificity of 96.0%, a positive predictive value of 98.1% and a negative predictive value of 96.0%.

Since the age variation is larger in the breast cancer group in the validation dataset, the concentration of these three metabolites in younger breast cancer patients and older breast cancer patients were analyzed. The result showed no significant difference for these three metabolites (lysoPC a C16:0, PC ae C42:5 and PC aa C34:2) between breast cancer patients with an age less than 41 years old (*n* = 17) and breast cancer patients with an age more than 60 years old (*n* = 12). Additionally, no significant difference was observed for these three metabolites with patients younger than 50 years old (*n* = 29) and older than 50 years old (*n* = 24). Therefore, it appears that age is not a confounding factor for these three metabolites.

### 2.4. Phosphatidylcholines

Glycerophospholipids contain two subclasses of lipid metabolites, PC diacyl (PC aa) and PC acyl-alkyl (PC ae). A total of 23 glycerophospholipids were selected as differentiating metabolites (Table 2), most of which were lower in the plasma of breast cancer patients (five of nine PC aas and 11 of 14 PC aes). PC ae 40:3 was detected as the most statistically significant decreased lipid among all the phosphatidylcholines (*p*-value of 2.79 × 10^−10^). The decreased glycerophospholipids in the plasma samples of breast cancer patients may reflect a higher activity of phospholipase A2 (PLA_2_), a family of enzymes, which hydrolyze glycerophospholipids to lysoPCs and fatty acids [18].

Previous studies revealed that the expression of PLA_2_ in cancer patients was upregulated [19,20]. The activity of PLA_2_ was observed as significantly higher in invasive breast tumor tissues than benign breast tumor tissues and normal breast tissues [21,22]. PLA_2_ is a diverse family of enzymes with different isoforms expressed across different tissues. Some specific classes of PLA_2_ were reported to be highly associated with breast cancer, such as sPLA_2_ (group IIA PLA_2_) [21,22] and PAF-AH (group VII and VIII PLA_2_) [23].

PLA_2_ can participate in the development of cancer through multiple mechanisms. It may mediate carcinogenesis by releasing lysoPCs, inducing cell growth via their metabolism to lysophosphatidic acid [24]. Some fatty acids, especially arachidonic acid, are released as well and metabolized into several molecules, many of which may induce cell growth and proliferation [25]. In our study, the concentrations of most lysoPCs were significantly decreased in the plasma samples of breast cancer patients, suggesting arachidonic acid metabolism might be a major contributor to breast cancer development. In addition, PLA_2_ may generate inflammatory mediators, which promote tumor formation [26]. Recently, PLA_2_ inhibitors have become attractive anti-cancer targets for the role of PLA_2_ in glycerophospholipid metabolism and carcinogenesis [27].

### 2.5. Lysophosphatidylcholines

LysoPCs were derived from partial hydrolysis of glycerophospholipids by phospholipase. In the identified differentiating metabolites between breast cancer patients and healthy controls, 10 of the selected metabolites were lysoPCs. The concentrations of the differentiating lysoPCs were detected to be lower in breast cancer patients compared to healthy controls. LysoPC a C16:0 and lysoPC a C18:0 were identified as the top two differentiating metabolites with the lowest *p*-values (1.21 × 10^−13^ for lysoPC a C16:0 and 4.21 × 10^−13^ for lysoPC a C18:0) across all the 39 differentiating metabolites. Interestingly, phosphatidylcholines stearic acid (C18:0) extracted from breast cancer tissue samples was previously reported to be significantly lower in those patients with developed metastasis and was even considered to be an independent intra-tumor marker of breast cancer prognosis [28]. Decreased levels of lysoPC a C16:0 and lysoPC a C18:0 were reported to be associated with a decrease PC level in hepatocellular carcinoma tissues [29].

These two lysoPCs (lysoPC a C 16:0 and lysoPC a C18:0) are among the most abundant lysoPCs in plasma. The decreased levels of two lysoPCs would largely contribute to the lower concentration of total lysoPCs in plasma, which was previously reported to be associated with an activated inflammatory status in cancer patients [30]. In fact, the total plasma lysoPCs (sum of the concentration of all the 14 lysoPCs detected in the current study) was observed to be significantly lower in breast cancer patients (*p* = 6.87 × 10^−8^, fold change (FC) = −1.67, breast cancer to healthy control). The mean concentration of lysoPC a C 20:4 demonstrated a decreasing trend from stage I to stage IV breast cancer patients (9.22 ± 3.62 μM for stage I, 8.64 ± 2.20 μM for stage II, 8.37 ± 3.20 μM for stage III and 7.87 ± 2.50 μM for stage IV). Those lysoPCs include very long chain fatty acids, such as lysoPC a C24:0, lysoPC a C26:0 and lysoPC a C28:0, which were also significantly lower in breast cancer patients. LysoPCs can be metabolized by lysophospholipase and further metabolized to fatty acids and choline. The lower levels of lysoPCs may reflect a higher metabolism rate in breast cancer patients. Hydrolysis of lysoPCs through lysophospholipase D derives lysophosphatidic acids, which are important lipid mediators that regulate cell proliferation and migration by binding G-protein-coupled receptors [31]. Autotaxin (a secreted lysophospholipase D) was reported to enhance tumor cell motility, survival and proliferation [32]. In fact, expression of autotaxin and lysophosphatidic acid was reported to contribute to the initiation and progression of breast cancer in a mice model [33].

### 2.6. Acylcarnitines

The higher hydrolysis rate of glycerophospholipids was associated with a higher level of acylcarnitines in the plasma sample of breast cancer patients compared with healthy controls. In this study, acylcarnitine C4 was selected as a differentiating metabolite. In addition, several other acylcarnitines (such as acylcarnitines C18, C18:2, C3 and C5) were detected at a higher concentration in breast cancer patients with *p*-values lower than 0.05 (*p* = 0.034 for C18, 0.037 for C18:2 and 0.015 for both C3 and C5). The increased acylcarnitine concentration indicates higher fatty acid beta-oxidation in breast cancer patients, which is consistent with recent studies that indicate that lipolysis and lipid oxidation are upregulated in cancer cells [34,35]. Fatty acids can be consumed through beta-oxidation to provide key substitute energy for cancer cell survival [36]. In some types of cancers, such as prostate cancer, fatty acid oxidation was proposed to be a dominant bioenergetic pathway [34]. The increased concentration of several acylcarnitines was also observed in kidney cancer patients and in those patients with high cancer grades, suggesting that acylcarnitine concentration could be a promising marker of cancer status and tumor grade [37].

### 2.7. Sphingomyelins

All five differentiating sphingomyelins (SMs), including SM (OH) C22:2, SM (OH) C14:1, SM (OH) C16:1, SM (OH) C22:1 and SM C20:2, were detected at higher levels in breast cancer patients. SM is an abundant constituent of cellular membranes [38] and is preferentially concentrated in the outer leaflet of the plasma membrane of most mammalian cells. SM plays an important role in the regulation of cell growth and differentiation through the SM cycle [39]. Some signaling metabolites, such as ceramides, sphingosines and sphingosine 1-phosphate (S1P), are involved in the cycle [40]. Many studies suggest that ceramide is an important signaling molecule in the cancer cells’ apoptotic response to inducers, such as the FAS/FAS ligand, tumor-necrosis factor-α (TNFα), growth factor withdrawal, hypoxia, hyperthermia and DNA damage [41]. In fact, higher levels of ceramide synthases and ceramide were detected in breast cancer tissues compared with those in adjacent normal ones [42]. S1P is another biologically active lipid derived from SM, which is reported to regulate cancer cell growth, survival and migration through both intracellular and receptor-mediated mechanisms [43]. Recently, Nagahashi and colleagues showed the importance of sphingosine kinase 1-produced S1P to breast cancer-induced hemangiogenesis and lymphangiogenesis in a mouse model [44]. Our results of increased circulating levels of the SMs may be an indication of dysfunction of the SM cycle in breast cancer patients, which may regulate cancer cell growth and metastasis.

Interestingly, all five sphingomyelins containing hydroxyl fatty acids were significantly higher in breast cancer patients (four were selected as differentiating metabolites, the other one was SM (OH) C24:1 with a *p*-value of 0.014 and an adjusted *q*-value of 0.050). Although the accurate position of the hydroxyl group in the fatty acids was unclear by this analysis, 2-hydroxy fatty acid has been widely reported in mammalian sphingomyelins (see review [45]). The fatty acid was catalyzed by fatty acid 2-hydroxylase and converted to 2-hydroxy fatty acid (hFA) and then incorporated into hFAceramide and complex hFA-sphingolipids [45]. A recent study showed that upregulation of fatty acid 2-hydroxylase could inhibit dibutyryl-cAMP-induced cell cycle exit in D6P2T Schwannoma cells [46]. The higher levels of hFA-sphingolipids indicate a higher expression level of fatty acid 2-hydroxylase, which may affect cancer cell growth in breast cancer patients.

**Figure 4:**
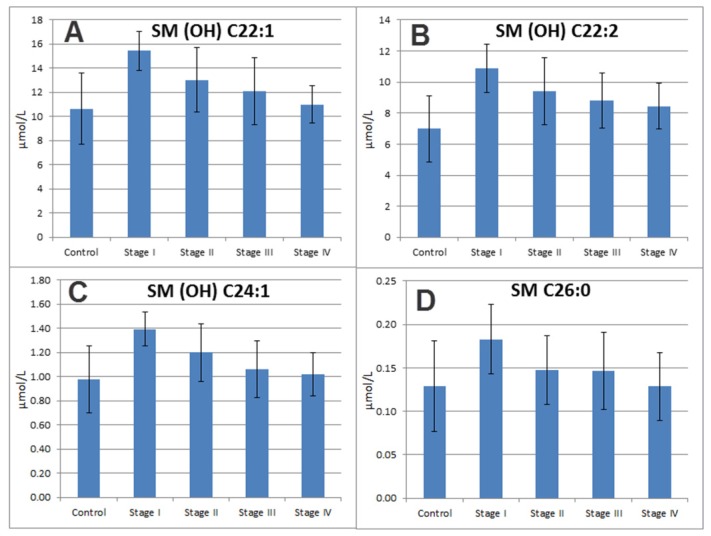
Bar plots of four characteristic sphingomyelins showed an elevated level in plasma samples in stage I caner as compared to controls and a decreasing trend in the plasma samples from stage I to stage IV breast cancer patients. (**A**) SM (OH) C22:1; (**B**) SM (OH) C22:2; (**C**) SM (OH) C24:1; and (**D**) SM C 26:0.

The concentrations of some SMs, such as SM (OH) C22:1, SM (OH) C22:2, SM (OH) C24:1 and SM C26:0, were detected to be higher in the early stages of breast cancer and showed a continuous decrease from stage I to stage IV breast cancer patients (Figure 4). SM (OH) C22:1, SM (OH) C24:1 and SM C26:0 were only significantly elevated in those breast cancer patients with early stage cancer (stage I and stage II, with a *p*-value (Student’s *t*-test) of 2.13 × 10^−4^, 2.28 × 10^−4^ and 0.03, respectively), but not in breast cancer patients at advanced stages (stage III and stage IV, with a *p*-value higher than 0.05) compared with healthy controls. These results indicate that some SMs markers are more affected in early stage patients. To the best of our knowledge, the underlying mechanism for decreased levels of those SMs in late stage patients compared to early stage patients remains to be elucidated.

## 3. Experimental Section

### 3.1. Sample Information

A total of 53 breast cancer patients and 25 healthy controls were collected from the City of Hope Comprehensive Cancer Center. To avoid the influence of age difference between breast cancer (BC) patients and healthy controls, the samples were divided into a training group and a testing group. In the training group, the average age is 41.3 (25–56) for the breast cancer group (30 subjects) and 38.2 (28–40) for the healthy control group (20 subjects), with no significant difference in age between the two groups (*p* = 0.111, Student’s *t*-test). More detailed information is provided in Table 2. To avoid the influence of food, all the blood samples were obtained before breakfast. The protocol was approved by the Institutional Review Board from the City of Hope Comprehensive Cancer. All participants signed informed consent before they were recruited for the study.

### 3.2. Sample Treatment and Metabolite Analysis

Our targeted metabolomics approach was based on electrospray ionization mass spectrometry (ESI-MS/MS) measurements using the AbsoluteIDQTM p180 kit (BIOCRATES Life Sciences AG, Innsbruck, Austria). The kit allows simultaneous quantification of 40 acylcarnitines (Cx:y), 90 glycerophospholipids (14 lysophosphatidylcholines (lyso PCx:y) and phosphatidylcholines (38 PCaa x:y and 38 PC ae x:y)), 15 sphingolipids (SMx:y or SM (OH)x:y) and 1 hexose. Cx:y represents the lipid side chain composition, where x indicates the number of carbons in the side chain, while y indicates the number of unsaturated chains. There are two types of side chain bonds in the glycerol moiety in the glycerophospholipids, ester (a) and ether (e). PC aa indicates that glycerol is bound to two fatty acid residues, while PC ae indicates the presence of a fatty acid residue and a fatty alcohol residual. The assay procedures and quality controls followed the user kit’s manufacturer instructions. The samples were analyzed on a 4000 QTRAP mass spectrometer (AB science) coupled with an Agilent 1200 series HPLC.

### 3.3. Data Analysis

Quantification of metabolite concentrations and quality assessment was performed with the MetIQ software package (BIOCRATES Life Sciences AG, Innsbruck, Austria). Internal standards serve as the reference for the metabolite concentration calculations. An xls file was exported, which contained sample names, metabolite names and metabolite concentration with the unit of μmol/L of plasma. The data was imported into SIMCA-P software (SIMCA-P 12.0, Umetrics, Umeå, Sweden) for multivariate analysis. Principle component analysis (PCA) and orthogonal partial least squares-discriminant analysis (OPLS-DA) were carried out to discriminate the metabolic patterns between breast cancer patients and healthy controls after mean centering and unit variance scaling. PCA is an unsupervised statistical analysis used to describe associations and patterns among a set of variables. R2X and Q2 are two measures of PCA model quality. R2X is the goodness of fit, which is the sum of squares of the entries of *X* explained by all extracted components. Q2 is the predictive power of the model, which is the fraction of the total variation of the entries of *X* that can be predicted by all extracted components, as estimated by cross validation. To guard against model over-fitting, the default 7-fold cross-validation was applied. The variable importance in the projection (VIP) values of all the metabolites from the 7-fold cross-validated OPLS-DA model were taken as a criterion for differentiating the metabolite selection. Those variables with VIP > 1.0 were selected as relevant for group discrimination [47]. Additionally, the nonparametric univariate method, the Student’s *t*-test, was applied to all metabolites. A classical one stage method of false discovery rate (FDR) was performed to adjust the *p*-value [48,49]. Differentiating metabolites were selected with VIP > 1 and *p* < 0.05 (adjusted *p*-value). The corresponding up- and down-regulated trend (fold change) showed how these selected differentiating metabolites varied between breast cancer patients and healthy controls and were used for subsequent metabolic pathway analysis. To further simplify the diagnostic model, all the selected differentiating metabolites were imported to SPSS software (v20, IBM, Chicago, IL, USA) for logical regression. Binary logical regression was performed with a forward method. Scatter plots were used to visualize the *y*-values calculated from the established equation in the SPSS software (v20, IBM, Chicago, IL, USA).

To further investigate the association between metabolite concentration and the pathological stages of breast cancer, all the samples in the training dataset and validation dataset were pooled together to enlarge the sample size (especially for stage I and stage IV patients). A Student’s *t*-test was performed between patients with early stages or advanced stages of breast cancer and the healthy controls. Bar plots were used to visualize the differences of the mean value of breast cancer patients at each stage and healthy controls using the Microsoft Excel package. Standard derivations of those metabolites in each stage were used to show the variation within each group.

## 4. Conclusions

In this study, we analyzed a panel of lipids in plasma samples from 55 breast cancer patients and 25 healthy controls using a targeted and quantitative metabolomics approach. The OPLS-DA model showed a distinct separation in lipid profiles between breast cancer patients from healthy controls using a training dataset composed of 30 patients and 20 healthy controls and successfully predicted breast cancer cases *versus* controls in the validation dataset. The selected differentiating metabolites (including 39 lipids) revealed lower levels of lysophosphatidylcholines and higher levels of sphingomyelins and acylcarnitines in breast cancer patients. A diagnostic equation using three metabolites (lysoPC a C16:0, PC ae C42:5 and PC aa C34:2) was established, which successfully separated breast cancer patients from healthy controls with a sensitivity of 98.1% and a specificity of 96.0%. One limitation for the current study is that the samples size is relatively small, especially for the healthy controls in the validation dataset. We will collect more samples to validate the diagnostic equation in our future work.

## Figures and Tables

**Table 1:** Differentiating metabolites between breast cancer patients and healthy controls identified from the learning dataset.

NO	Metabolites	Classes	VIP a	FC b	*p*-value c	*q*-value d
1	PC ae C40:3	Phosphatidylcholines	2.31	−4.24	2.79 × 10^−10^	1.36 × 10^−8^
2	PC aa C42:4	Phosphatidylcholines	2.09	−1.96	4.87 × 10^−8^	9.73 × 10^−7^
3	PC ae C38:3	Phosphatidylcholines	2.09	−2.15	5.16 × 10^−8^	9.73 × 10^−7^
4	PC ae C40:4	Phosphatidylcholines	2.08	−1.93	5.33 × 10^−8^	9.73 × 10^−7^
5	PC ae C38:1	Phosphatidylcholines	2.05	−72.18	1.16 × 10^−7^	1.82 × 10^−6^
6	PC ae C42:4	Phosphatidylcholines	1.88	−1.63	2.08 × 10^−6^	2.76 × 10^−5^
7	PC ae C40:5	Phosphatidylcholines	1.86	−1.63	2.73 × 10^−6^	3.32 × 10^−5^
8	PC ae C42:5	Phosphatidylcholines	1.83	−1.44	4.68 × 10^−6^	5.25 × 10^−5^
9	PC aa C40:2	Phosphatidylcholines	1.82	−2.24	5.63 × 10^−6^	5.87 × 10^−5^
10	PC ae C44:3	Phosphatidylcholines	1.78	−1.63	9.07 × 10^−6^	8.83 × 10^−5^
11	PC ae C38:2	Phosphatidylcholines	1.67	−1.84	4.31 × 10^−5^	3.93 × 10^−4^
12	PC ae C42:1	Phosphatidylcholines	1.66	−1.63	4.69 × 10^−5^	4.02 × 10^−4^
13	PC aa C40:3	Phosphatidylcholines	1.65	−1.62	5.43 × 10^−5^	4.40 × 10^−4^
14	PC ae C36:2	Phosphatidylcholines	1.56	1.39	1.64 × 10^−4^	1.14 × 10^−3^
15	PC aa C38:6	Phosphatidylcholines	1.48	1.46	3.64 × 10^−4^	2.21 × 10^−3^
16	PC ae C40:6	Phosphatidylcholines	1.36	1.31	1.22 × 10^−3^	7.10 × 10^−3^
17	PC aa C38:0	Phosphatidylcholines	1.31	1.44	1.92 × 10^−3^	1.08 × 10^−2^
18	PC ae C34:2	Phosphatidylcholines	1.26	1.33	2.89 × 10^−3^	1.51 × 10^−2^
19	PC aa C40:6	Phosphatidylcholines	1.26	1.36	2.90 × 10^−3^	1.51 × 10^−2^
20	PC ae C40:2	Phosphatidylcholines	1.17	−1.32	6.22 × 10^−3^	2.93 × 10^−2^
21	PC aa C40:4	Phosphatidylcholines	1.16	−1.38	6.71 × 10^−3^	2.97 × 10^−2^
22	PC aa C34:2	Phosphatidylcholines	1.14	1.19	7.98 × 10^−3^	3.43 × 10^−2^
23	PC aa C42:5	Phosphatidylcholines	1.13	−1.32	8.63 × 10^−3^	3.60 × 10^−2^
24	lysoPC a C16:0	Lysophosphatidylcholines	2.53	−1.98	1.21 × 10^−13^	1.77 × 10^−11^
25	lysoPC a C18:0	Lysophosphatidylcholines	2.5	−2.25	4.21 × 10^−13^	3.08 × 10^−11^
26	lysoPC a C20:4	Lysophosphatidylcholines	2.2	−2.14	4.04 × 10^−9^	1.48 × 10^−7^
27	lysoPC a C18:1	Lysophosphatidylcholines	2.11	−1.88	3.30 × 10^−8^	9.63 × 10^−7^
28	lysoPC a C17:0	Lysophosphatidylcholines	2.04	−1.73	1.25 × 10^−7^	1.82 × 10^−6^
29	lysoPC a C20:3	Lysophosphatidylcholines	1.6	−1.63	1.03 × 10^−4^	7.52 × 10^−4^
30	lysoPC a C28:0	Lysophosphatidylcholines	1.16	−1.23	6.63 × 10^−3^	2.97 × 10^−2^
31	lysoPC a C16:1	Lysophosphatidylcholines	1.09	−1.3	1.09 × 10^−2^	4.32 × 10^−2^
32	lysoPC a C24:0	Lysophosphatidylcholines	1.09	−1.2	1.09 × 10^−2^	4.32 × 10^−2^
33	lysoPC a C26:0	Lysophosphatidylcholines	1.08	−1.28	1.17 × 10^−2^	4.38 × 10^−2^
34	SM (OH) C22:2	Sphingomyelins	1.62	1.38	8.26 × 10^−5^	6.35 × 10^−4^
35	SM (OH) C14:1	Sphingomyelins	1.52	1.37	2.40 × 10^−4^	1.59 × 10^−3^
36	SM (OH) C16:1	Sphingomyelins	1.49	1.34	3.49 × 10^−4^	2.21 × 10^−3^
37	SM (OH) C22:1	Sphingomyelins	1.2	1.23	5.07 × 10^−3^	2.55 × 10^−2^
38	SM C20:2	Sphingomyelins	1.08	1.32	1.17 × 10^−2^	4.38 × 10^−2^
39	C4	Acylcarnitines	1.18	1.45	5.64 × 10^−3^	2.74 × 10^−2^

a Variable importance in the projection (VIP) was obtained from OPLS-DA with a threshold of 1.0;

b The fold change (FC) was calculated by the average value of the breast cancer group to that of the control group. FC with a value larger than zero indicates a higher level of the plasma metabolite in breast cancer patients, while a FC value lower than zero indicates a lower level, compared to healthy controls;

c *p*-values are calculated from a Student’s *t*-test;

d *q*-values are the adjusted *p*-value with the false discovery rate (FDR).

**Table 2:** Clinical information and characteristics of human subjects. BC, breast cancer.

	Training group		Validation group	
	
	Control (*n* = 20)	BC (*n* = 30)	Control (*n* = 5)	BC (*n* = 23)
Age (mean, range)	38.2 (28–40)	41.3 (25–56)	34.8 (21–39)	56.2 (40–67)
Stage				
TNM-I	/	4	/	4
TNM-II	/	11	/	8
TNM-III	/	11	/	7
TNM-IV	/	4	/	4
